# Multiple Intestinal Bacteria Associated with the Better Protective Effect of *Bifidobacterium pseudocatenulatum* LI09 against Rat Liver Injury

**DOI:** 10.1155/2022/8647483

**Published:** 2022-01-28

**Authors:** Hua Zha, Guinian Si, Chenyu Wang, Hua Zhang, Lanjuan Li

**Affiliations:** ^1^State Key Laboratory for Diagnosis and Treatment of Infectious Disease, Collaborative Innovation Center for Diagnosis and Treatment of Infectious Diseases, National Clinical Research Center for Infectious Diseases, The First Affiliated Hospital, Zhejiang University School of Medicine, China; ^2^Department of Rehabilitation, Shulan (Hangzhou) Hospital, Zhejiang Shuren University School of Medicine, China

## Abstract

*Bifidobacterium pseudocatenulatum* LI09 could protect rats from D-galactosamine- (D-GalN-) induced liver injury. However, individual difference in the protective effects of LI09 on the liver injury remains poorly understood. The present study is aimed at determining the multiple intestinal bacteria associated with the better protective effect of LI09 against D-GalN-induced rat liver injury. Two rat cohorts, i.e., the nonsevere and severe cohorts, were divided based on their liver injury severity. Higher level of ALB and lower levels of ALT, AST, TBA, TB, IL-5, and MIP-3*α* were determined in the nonsevere cohort than the severe cohort. The alpha diversity indices (i.e., observed species, Shannon, and Pielou indices) did not yield significant differences between the intestinal microbiota of the nonsevere and severe cohorts. The intestinal microbiota composition was different between the two cohorts. Ten phylotypes assigned to *Bacteroides*, Clostridia_UCG-014, *Clostridium* Lachnospiraceae, Lachnospiraceae_NK4A136, and *Parabacteroides* were closely associated with the nonsevere cohort, among which, ASV8_Lachnospiraceae_NK4A136 was the most associated one. At the structure level, two groups of phylotypes with most correlations were determined in the intestinal microbiota networks of the two cohorts. Among them, ASV135_Lachnospiraceae_NK4A136 was the most powerful gatekeeper in the microbiota network of the nonsevere cohort. In conclusion, some intestinal bacteria, e.g., Lachnospiraceae_NK4A136, *Parabacteroides*, and *Clostridium*, were associated with the better protective effect of LI09 against D-GalN-induced rat liver injury. They were likely to enhance the effectiveness of LI09, and their clinical application deserves further investigation.

## 1. Introduction

Liver injury could result in critical illness and even mortality in human beings [1, 2]. The progression of liver injury is associated with the alterations of the gut microbiota [3, 4]. A variety of materials and products are capable of preventing liver diseases via modulating the gut microbiota in the individuals or animal models [5–8], while some alternative factors, e.g., hormone, were also associated with the prevention of liver diseases [9].

Probiotic bacteria were determined with protective effects against different kinds of liver injury [10]. *Lactobacillus buchneri* TCP016 could alleviate the liver injury induced by LPS and D-galactosamine (D-GalN) via reshaping the intestinal microbiota [11]. *Pediococcus pentosaceus* PP04 was capable of modulating the gut inflammation and intestinal microbiota to relieve the mice liver injury induced by high-fat diet [12]. *Bifidobacterium longum* LC67 and *Lactobacillus plantarum* LC27 could restore the disturbance of gut microbiota in mice to attenuate the liver damage induced by 2,4,6-trinitrobenzesulfonic acid [13].

The prevention of D-GalN-induced rat liver injury by probiotics has been previously conducted [14, 15]. *Bifidobacterium pseudocatenulatum* LI09 was originally isolated from the feces of healthy individual and was capable of alleviating D-GalN-induced rat liver injury [16]. However, the intestinal bacterial microbiota associated with the individual differences in the liver injury severity of LI09-pretreated rats remain poorly understood. The present study was designed to determine the multiple intestinal bacteria associated with the better protective effect of LI09 against D-GalN-induced rat liver injury.

## 2. Materials and Methods

### 2.1. Animal Experiment

The procedures for animal experiment were as described by Fang et al. [16], with a few modifications. Briefly, LI09 was revived on the trypticase phytone yeast agar plate at 37°C for 36 h, before being prepared in physiological saline at a final concentration of 3 × 10^9^ CFU/mL. Forty pathogen-free male rats (Sprague-Dawley) weighting from 250 to 350 g were fed with a standard laboratory rat chow and exposed to a 12 : 12 light/dark cycle at 22°C. All the steps were conducted according to the 2011 National Institutes of Health Guide for Laboratory Animals.

Each of the 40 rats was orally administrated with a 1 mL aliquot of LI09 (3 × 10^9^ CFU) for one week. On the eighth day, the rats were given an intraperitoneal injection of D-GalN to induce liver injury at a dose of 700 mg/kg body weight. After 24 h, each of the alive rats received an anesthetization via an intraperitoneal injection of 10 mg/kg xylazine and 80 mg/kg ketamine and then subjected to laparotomy with an eventual unconscious death. The caecal content, blood, and liver samples were collected during the laparotomy. The protocols for the current study were approved by Animal Care and Use Committee of the First Affiliated Hospital, Zhejiang University School of Medicine.

### 2.2. Evaluation of Liver Injury Severity

The tissue from left liver lobe of all rats were fixed in 10% formalin solution and then dehydrated and processed in paraffin using standard histological methods. The liver samples were stained and mounted on microscope slides. The liver injury severity was evaluated by a professional pathologist based on the Ishak scoring system [17].

The LI09 rats were divided into two cohorts based on the Ishak score of each rat, i.e., “nonsevere” cohort (Ishak score < 10) and “severe” cohort (Ishak score ≥ 10).

### 2.3. Measurement of Liver Function Variables

Serum was extracted from blood samples by centrifugation, before being stored at -80°C for the subsequent experiments. Concentrations of liver function variables in serum, i.e., gamma-glutamyl transpeptidase (GGT), albumin (ALB), aspartate aminotransferase (AST), total bilirubin (TB), alanine aminotransferase (ALT), alkaline phosphatase (ALP), and total bile acid (TBA), were measured by an automatic biochemical analyser (Roche Diagnostics, Germany) according to the manufacturer's protocol.

### 2.4. Measurement of Serum Cytokines

The concentrations of 23 cytokines in all serum samples were measured using a Bio-Plex Pro™ Rat Cytokine 23-Plex Assay kit (Bio-Rad Ltd., USA) as per the instructions of the manufacturer. These cytokines included macrophage inflammatory protein- (MIP-) 1*α*, MIP-3*α*, granulocyte/macrophage colony-stimulating factor (GM-CSF), granulocyte colony-stimulating factor (G-CSF), regulated upon activation, normal T cell expressed and secreted (RANTES), interferon- (IFN-) *γ*, tumor necrosis factor- (TNF-) *α*, interleukin- (IL-) 1*α*, IL-1*β*, IL-2, IL-4, IL-5, IL-6, IL-7, IL-10, IL-12p70, IL-13, IL-17A, IL-18, macrophage colony-stimulating factor (M-CSF), monocyte chemoattractant protein-1 (MCP-1/CCL2), growth-regulated oncogene/keratinocyte chemoattractant (GRO/KC), and vascular endothelial growth factor (VEGF).

### 2.5. Molecular Experiments

DNA was extracted from the caecal samples by using a Dneasy Powersoil kit (MoBio Laboratories Inc., USA) according to the manufacturer's description and then amplified with the bacterial primers (i.e., 341F/785R). The PCR products were purified by using a DNA Clean and Concentrator Kit (Zymo Research, USA), and their concentrations were measured by using a Qubit™ dsDNA HS Assay Kit (Thermo Fisher Scientific Inc., USA). The purified PCR products were submitted to sequencing lab and sequenced on Illumina Novaseq 6000 platform (Illumina Inc. USA).

### 2.6. Processing of Sequencing Data

The standard bioinformatics procedures were used to process the sequencing data. Briefly, the sequencing data were imported and processed with QIIME2 [18]. The procedures, i.e., quality filter, denoise, and merge were conducted using the DADA2 plugin to generate the amplicon sequence variant (ASV) table [19]. Chimera and singletons were removed from the dataset. Taxonomy was assigned to all the filtered ASVs against the Silva 138 database in QIIME2 [20]. The sample reads were rarefied for the following analyses.

### 2.7. Microbiota Composition Analyses

The alpha diversity indices, i.e., observed species, Shannon, and Pielou indices, between the nonsevere and severe cohorts were calculated. Permutational analysis of variance (PERMANOVA) was performed in R 4.1.0 to compare the microbiota composition of the nonsevere and severe cohorts. Similarity percentage (SIMPER) analysis was carried out to compare the microbiota dissimilarity of the nonsevere and severe cohorts, as well as the phylotypes contributing to the dissimilarity. Nonmetric multidimensional scaling (nMDS) was used to visualise the intestinal microbiota in the nonsevere and severe cohorts. Linear discriminant analysis effect size (LEfSe) was conducted to determine the phylotypes associated with the nonsevere or severe cohort. Representative phylotypes in the nonsevere and severe cohorts were defined as the ASVs associated with the nonsevere or severe cohorts determined by both SIMPER and LEfSe analyses.

A Spearman test was carried out to determine the correlations between the representative phylotypes in the nonsevere cohort and the clinical variables with different levels between the nonsevere and severe cohorts.

### 2.8. Microbiological Network Analyses

Cooccurrence network inference (CoNet) program was used to investigate the correlations between the phylotypes in the microbiota networks of the nonsevere and severe cohorts. Five metrics, i.e., Pearson, Spearman, mutual information, Bray-Curtis, and Kullback-Leibler dissimilarities, were used to calculate the ensemble inference in the nonsevere and severe cohorts. The 10 ASVs with most correlations in the networks of the nonsevere and severe cohorts were demonstrated.

Network fragmentation calculations and generation of a null distribution were performed in R to explore the network gatekeeper(s) in the nonsevere and severe cohorts. Statistical significance was defined as the number of times a fragmentation score over that resulting from the removal of the phylotype observed within the null distribution.

### 2.9. Statistical Analyses

*t*-tests were performed to compare the nonsevere and severe cohorts for (1) the liver function variables ALB and ALP; (2) the cytokine variables IL-1*α*, MCP-1/CCL2, IL-4, IL-10, IL-12p70, IL-17A, IL-18, M-CSF, MIP-1*α*, and RANTES; and (3) the alpha diversity indices, i.e., observed species, Shannon, and Pielou indices. The liver function variables ALT, AST, and TBA and cytokine variables G-CSF, IL-2, IL-13, GRO/KC, and VEGF in the nonsevere and severe cohorts were transformed in square root and compared with *t*-tests. The liver function variable TB and cytokine variables GM-CSF, IFN-*γ*, IL-6, and IL-7 in the two cohorts were transformed in log10 before being compared with *t*-tests. Mann–Whitney tests were used to compare the two cohorts for the liver function variable GGT and cytokine variables IL-1*β*, IL-5, MIP-3*α*, and TNF-*α*.

## 3. Results

### 3.1. Evaluation of Liver Injury Severity

A total of 24 were determined with Ishak scores < 10, and the remaining 16 had Ishak scores ≥ 10. Therefore, 24 rats were classified as “nonsevere” cohort, while 16 rats were as “severe” cohort for the present study.

### 3.2. Analyses of Clinical Variables

**Figure 1:**
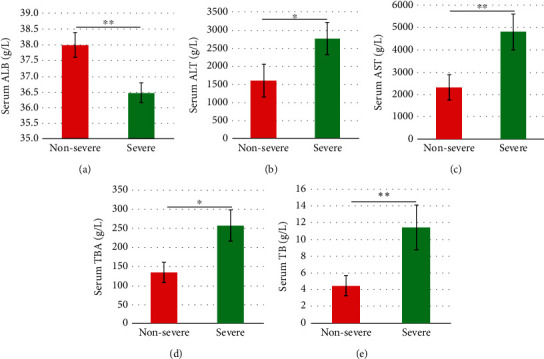
Statistical analyses showed the differences in liver function variables, i.e., (a) ALB, (b) ALT, (c) AST, (d) TBA, and (e) TB, between the *Bifidobacterium pseudocatenulatum* LI09-pretreated rats with two different levels of D-GalN-induced liver injury, i.e., the nonsevere and severe cohorts. ^∗^*P* value between 0.01 and 0.05, while ^∗∗^*P* value less than 0.01.

Five out of the seven liver function variables were determined with different concentrations between the nonsevere and severe cohorts (Figure 1). ALB was greater in the nonsevere cohort than the severe cohort (Figure 1(a)), while ALT, AST, TBA, and TB were at greater levels in the severe cohort than the nonsevere cohort (Figures 1(b)–1(e)).

**Figure 2:**
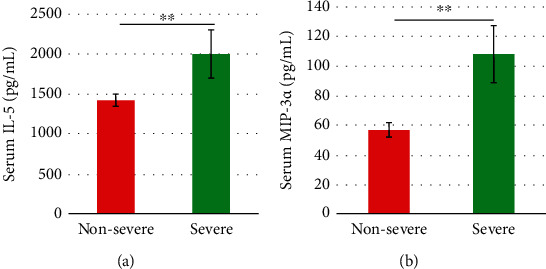
Statistical analyses showed the differences in cytokine variables, i.e., (a) IL-5 and (b) MIP-3*α*, between the nonsevere and severe cohorts. ^∗∗^*P* value less than 0.01.

The majority of cytokines (i.e., 21 out of 23) were determined with similar concentrations in the nonsevere and severe cohorts (all *P* > 0.05). IL-5 and MIP-3*α* were both at lower concentrations in the nonsevere cohort and severe cohort (Figures 2(a) and 2(b)).

### 3.3. Microbiota Composition Analyses

**Figure 3:**
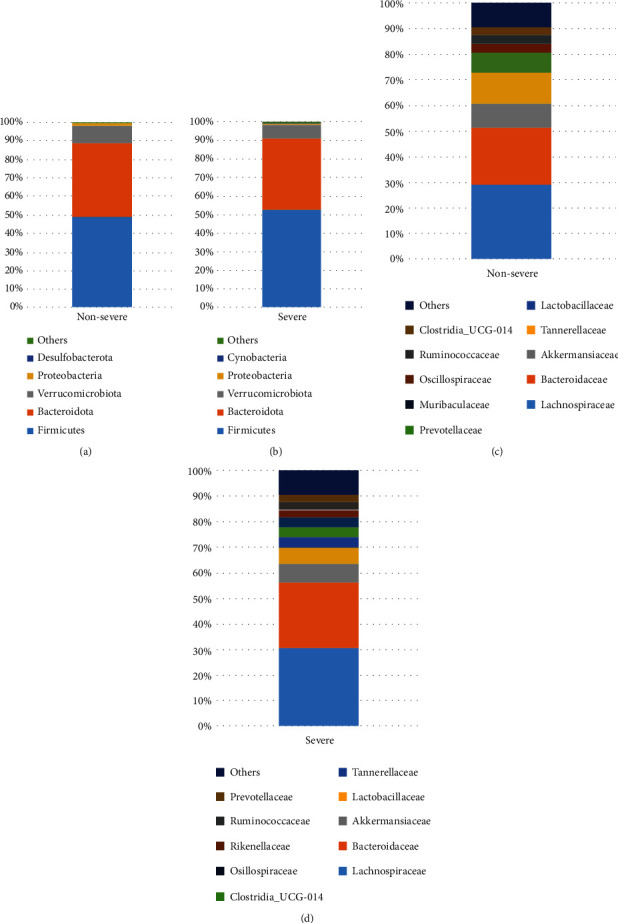
Microbiota composition in the nonsevere and severe cohorts. (a, b) The phyla in the microbiota of the nonsevere and severe cohorts, respectively. (c, d) The families in the microbiota of the nonsevere and severe cohorts, respectively. Note: only the top five phyla and top 10 families of the cohorts were demonstrated in the figure.

The four phyla, i.e., Firmicutes, Bacteroidota, Verrucomicrobiota, and Proteobacteria, accounted for the majority of microbiota composition in both the nonsevere and severe cohorts (Figures 3(a) and 3(b)). At the family level, Lachnospiraceae, Bacteroidaceae, Akkermansiaceae, and Tannerellaceae constituted the most of microbiota composition in both of the two cohorts (Figures 3(c) and 3(d)).

**Figure 4:**
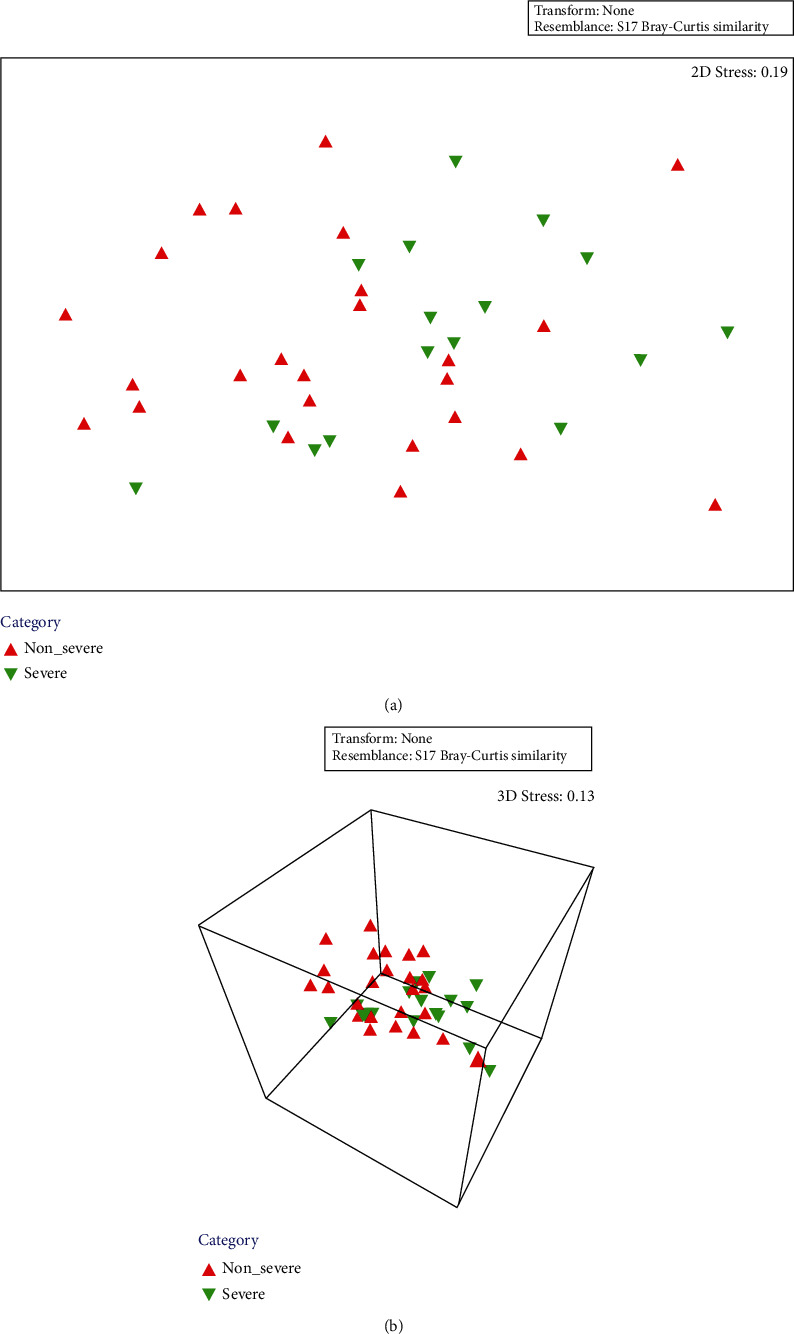
The intestinal microbiota in the nonsevere and severe cohorts visualised by nonmetric multidimensional scaling analysis in (a) 2D and (b) 3D.

Observed species, Shannon, and Pielou indices were all similar between the nonsevere cohort and severe cohort (Supplemental Table S1). PEMANOVA results indicated a significant difference in the microbiota composition between the two cohorts (*R*^2^ = 0.049, *P* < 0.02). nMDS plot also showed a difference in the intestinal microbiota between the nonsevere and severe cohorts (Figure 4). SIMPER results showed a difference in the microbiota between the two cohorts (SIMPER average dissimilarity = 51.8%). The microbiota similarity within the nonsevere cohort was slightly greater than that within the severe cohort (SIMPER average similarity, 50.2% versus 48.7%).

**Figure 5:**
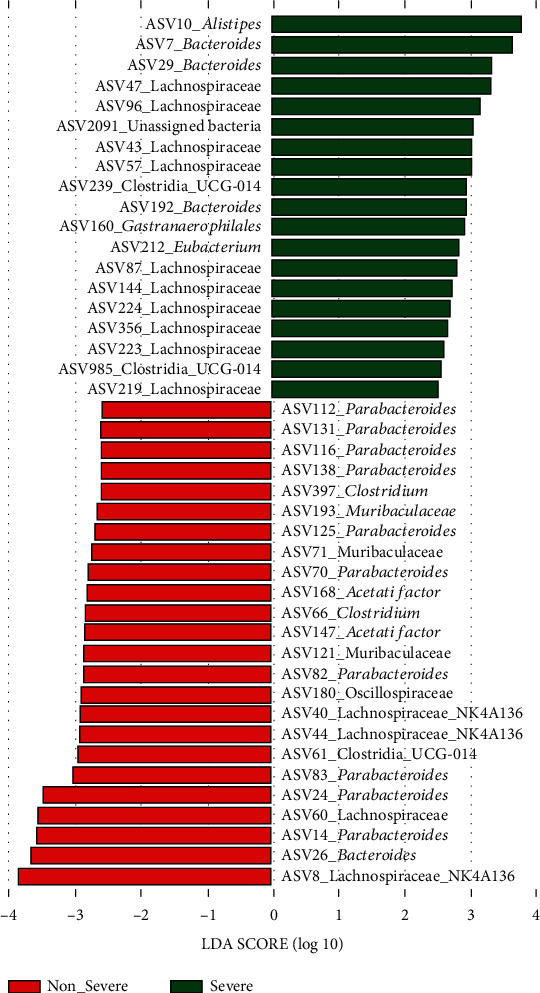
Linear discriminant analysis effect size determined the phylotypes associated with the nonsevere and severe cohorts.

A total of 96 ASVs contributed to the dissimilarity between the nonsevere and severe cohorts, among which, 49 ASVs were associated with the nonsevere cohort, while 47 ASVs were associated with the severe cohort (Supplemental Table S2). LEfSe analysis determined 24 ASVs associated with the nonsevere cohort and 19 ASVs associated with the severe cohort (Figure 5).

A total of 10 ASVs assigned to *Bacteroides*, Clostridia_UCG-014, *Clostridium* Lachnospiraceae, Lachnospiraceae_NK4A136, and *Parabacteroides* were associated with the nonsevere cohort according to both SIMPER and LEfSe results (Table 1(a)), among which, ASV8_Lachnospiraceae_NK4A136 was most associated with the nonsevere cohort. Likewise, ten phylotypes were determined with most associations with the severe cohort (Table 1(b)), and ASV10_*Alistipes* was the most associated one.

**Figure 6:**
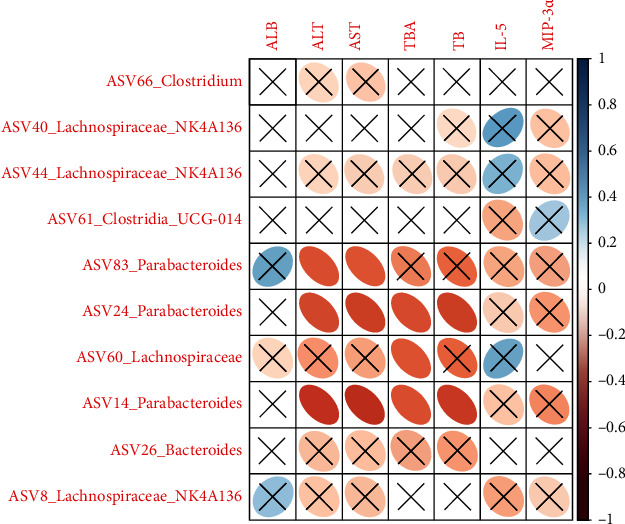
Correlations between the bacterial phylotypes associated with the nonsevere cohort and the clinical variables at different levels between the nonsevere and severe cohorts. Note: the scale bar represented the correlation coefficient, and only those over 0.4 (or less than -0.4) with a significant difference (*P* value < 0.05) were showed without a cross-label.

ASV83_*Parabacteroides*, ASV24_*Parabacteroides*, ASV60_Lachnospiraceae, and ASV14_*Parabacteroides* were negatively correlated with ALT, AST, TBA, and/or TB (Figure 6).

### 3.4. Microbiological Network Analyses

CoNet results showed 10 phylotypes assigned to Lachnospiraceae_NK4A136, Muribaculaceae, Prevotellaceae_NK3B31, Lachnospiraceae, and Oscillospiraceae had most correlations in the microbiota network of the nonsevere cohort (Table 2). A total of seven gatekeepers were identified in the microbiota network of the nonsevere cohort (fragmentation analyses, all *P* < 0.05) (Table 2), among which, ASV135_Lachnospiraceae_NK4A136 were determined as the most powerful structural gatekeeper with the most correlations in the nonsevere cohort (fragmentation analyses, all *P* < 0.005).

Likewise, 10 phylotypes with most correlations in the severe cohort were determined by CoNet (Table 2), six of which were determined as structural gatekeepers in the microbiota network (fragmentation analyses, all *P* < 0.04) (Table 2). ASV351_*Oscillibacter* was determined as the most powerful gatekeeper in the microbiota network of the severe cohort (fragmentation analyses, all *P* < 0.004).

## 4. Discussion

*B. pseudocatenulatum* LI09 has been determined with protective effects against D-GalN-induced liver injury in rats [16]. In the current study, individual difference was determined in the protective effects of LI09 on D-GalN-induced rat liver injury based on the Ishak score results. Ishak scoring system has been used in multiple disease studies to evaluate the extent of liver pathology [21–23]. In the present study, we aimed to characterise the intestinal bacteria associated with the better protective effect of LI09 against liver injury.

The multiple liver function and cytokine variables were measured in multiple studies to achieve different objectives [12, 24, 25]. The higher level of ALB and lower levels of ALT, AST, TBA, TB, IL-5 and MIP-3*α* in the nonsevere cohort than the severe cohort could suggest the alterations of these variables were associated with better protective effects of LI09 against liver injury. Some of these variables were also determined in the LI09-pretreated rats with D-GalN-induced liver injury than the positive control (PC) cohort, i.e., ALT, AST, TBA, and MIP-3*α* [16]. Some alternative aspects (i.e., specific immune and liver function profiles) should be explored to benefit the potential personalized microbiome modification [26, 27].

Observed species, Shannon, and Pielou indices have been used for evaluating the alpha diversity of microbiota [28, 29]. In this study, no difference was determined in the three indices between the intestinal microbiota of the nonsevere and severe cohorts, suggesting there was no significant difference in alpha diversity between the two microbiota. PERMANOVA and SIMPER analyses have been performed in different studies to evaluate the difference between different microbiota [30, 31]. The two analyses showed the microbiota composition were different between the nonsevere and severe cohorts, suggesting the alterations of intestinal microbiota were associated with the different levels of liver injury severity in the LI09-treated rats.

SIMPER and LEfSe analyses have been performed to determine the representative phylotypes associated with the specific microbiota [28–30]. In the current study, 20 ASVs were associated with the nonsevere or severe cohort, among which, ASV8_Lachnospiraceae_NK4A136 and ASV10_*Alistipes* were most associated with the nonsevere and severe cohorts, respectively. Lachnospiraceae_NK4A136 was regarded as a beneficial intestinal taxon [32]. *Alistipes* has been determined with a tight correlation with the indicator of alcoholic liver disease [33], and in this study, it was more likely to contribute to the worse liver injury.

In addition to Lachnospiraceae_NK4A136, *Parabacteroides*, Clostridia_UCG-014, and *Clostridium* were also determined as the representative bacteria in the nonsevere cohort but not in the severe cohort. These bacteria were the potential biomarkers in the nonsevere cohort. *Parabacteroides* was determined with commensal intestinal bacteria with an antiobesity effect [34, 35]. The abundance of intestinal Clostridia_UCG-014 was found lower in the polycystic ovary syndrome cohort than the healthy control cohort [36]. *Clostridium* spp. were determined with both beneficial and harmful effects [37], and in this study, it was more likely to be beneficial.

The correlations between the intestinal bacteria and clinical variables have been investigated in different studies [38, 39]. Four representative phylotypes in the nonsevere cohort assigned to *Parabacteroides* and Lachnospiraceae were negatively correlated with ALT, AST, ABA, and/or TB, suggesting they were likely to reduce the these liver function and cytokine variables.

The microbiota networks have been investigated in multiple studies [40–42], as well as the structural gatekeepers [30, 43]. Based on the CoNet and fragmentation analyses, ASV135_Lachnospiraceae_NK4A136 were determined as the most powerful structural gatekeeper with most correlations in the nonsevere cohort. As mentioned above, another phylotype (i.e., ASV8_Lachnospiraceae_NK4A136) was most associated with the nonsevere cohort at the microbiota composition level, suggesting that Lachnospiraceae_NK4A136 could be a vital microbe in the intestinal microbiota of the nonsevere cohort.

There were still some limitations in this work. For example, the animal amount for the two probiotics pre-treated cohorts (i.e., 24 versus 16) was relatively limited. Although many conditions (e.g., sex, weight, housing temperature, and light) were controlled, some additional factors (e.g., animal characteristics) were not observed and recorded for further investigation. We acknowledge that these need to be improved for more convincing results in the future work.

In conclusion, some intestinal bacteria, e.g., Lachnospiraceae_NK4A136, *Parabacteroides*, and *Clostridium*, were associated with the better protective effect of LI09 against D-GalN-induced rat liver injury. Their potential clinical application for assisting LI09 in lowering the liver injury severity deserves further investigation.

## Figures and Tables

**(a):** 

Taxonomy	LDA score	Nonsevere Av.Abund	Severe Av.Abund	Contrib%
ASV8_Lachnospiraceae_NK4A136	3.82	1590	711	2.32
ASV26_*Bacteroides*	3.63	695	299	0.9
ASV14_*Parabacteroides*	3.56	797	414	0.83
ASV60_Lachnospiraceae	3.53	426	93	0.65
ASV24_*Parabacteroides*	3.45	564	264	0.59
ASV83_*Parabacteroides*	3.00	222	103	0.25
ASV61_Clostridia_UCG-014	2.92	213	99	0.32
ASV44_Lachnospiraceae_NK4A136	2.90	124	110	0.34
ASV40_Lachnospiraceae_NK4A136	2.89	128	118	0.35
ASV66_*Clostridium*	2.79	209	135	0.28

**(b):** 

Taxonomy	LDA score	Nonsevere Av.Abund	Severe Av.Abund	Contrib%
ASV10_*Alistipes*	3.78	1008	1507	2.42
ASV7_*Bacteroides*	3.63	968	1389	1.87
ASV29_*Bacteroides*	3.32	300	526	0.7
ASV47_Lachnospiraceae	3.30	114	353	0.5
ASV96_Lachnospiraceae	3.15	64	206	0.32
ASV43_Lachnospiraceae	3.02	75	171	0.28
ASV57_Lachnospiraceae	3.02	157	278	0.44
ASV160_*Gastranaerophilales*	2.91	25	114	0.21
ASV212_*Eubacterium*	2.82	55	150	0.29
ASV87_Lachnospiraceae	2.80	96	166	0.28

**Table 2:** The top 10 phylotypes with most correlations in the nonsevere cohort and severe cohort determined by cooccurrence network inference analysis. Note: ^∗^ represented the phylotypes identified as a gatekeeper in the microbiota network by fragmentation analysis.

Nonsevere cohort		Severe cohort	
ASV ID	Taxonomy	ASV ID	Taxonomy
ASV135^∗^	Lachnospiraceae_NK4A136	ASV405^∗^	Clostridia_UCG-014
ASV501^∗^	Muribaculaceae	ASV234^∗^	*Muribaculum*
ASV50^∗^	Muribaculaceae	ASV566^∗^	*Bacteroides*
ASV544^∗^	Muribaculaceae	ASV724^∗^	*Monoglobus*
ASV483	Muribaculaceae	ASV39	Muribaculaceae
ASV323^∗^	Lachnospiraceae	ASV503	Clostridia_UCG-014
ASV134^∗^	Prevotellaceae_NK3B31	ASV50	Muribaculaceae
ASV161^∗^	Lachnospiraceae	ASV519	Lachnospiraceae
ASV214	Oscillospiraceae	ASV351^∗^	*Oscillibacter*
ASV121	Muribaculaceae	ASV463^∗^	Clostridia_UCG-014

## Data Availability

The sequencing data for the current study are available in Sequence Read Archive (PRJNA755955).

## Supplementary Materials

Supplementary Materials Supplemental Table S1: comparisons of alpha diversity indices between nonsevere and severe cohorts. Supplemental Table S2: similarity percentage analysis determined the phylotypes associated with intestinal microbiota in (A) nonsevere and (B) severe cohorts.
