# Supplementary material for: Multiple Intestinal Bacteria Associated with the Better Protective Effect of Bifidobacterium pseudocatenulatum LI09 against Rat Liver Injury
Source: Biomed Res Int. 2022 Jan 28;2022:8647483. doi: 10.1155/2022/8647483 (PMC8816544; doi:10.1155/2022/8647483)
Supplement: Supplementary Materials — Supplemental Table S1: comparisons of alpha diversity indices between nonsevere and severe cohorts. Supplemental Table S2: similarity percentage analysis determined the phylotypes associated with intestinal microbiota in (A) nonsevere and (B) severe cohorts. [file 8647483.f1.docx]

**Supplemental materials**

Table S1. Comparisons of alpha diversity indices between Non-Severe and Severe cohorts. Note: The values were demonstrated in mean ± standard error.

| Alpha diversity index | Non-Severe cohort | Severe cohort |
| --- | --- | --- |
| Observed species | 437 ± 5 | 436 ± 9 |
| Shannon index | 4.51 ± 0.03 | 4.45 ± 0.09 |
| Pielou index | 0.74 ± 0.005 | 0.73 ± 0.01 |

Table S2. Similarity percentage analysis determined the phylotypes associated with intestinal microbiota in (A) Non-Severe and (B) Severe cohorts.

| A | Non-Severe cohort | Severe cohort |  |
| --- | --- | --- | --- |
| Species | Av.Abund | Av.Abund | Contrib% |
| ASV1 | 5548.71 | 4293.44 | 7.01 |
| ASV8 | 1589.79 | 710.63 | 2.32 |
| ASV12 | 1242.33 | 863.69 | 1.8 |
| ASV4 | 2307.46 | 1918.06 | 1.74 |
| ASV6 | 1402.88 | 1280.94 | 1.46 |
| ASV11 | 959.75 | 671.44 | 1.02 |
| ASV26 | 695.17 | 298.94 | 0.9 |
| ASV14 | 797.38 | 414 | 0.83 |
| ASV31 | 584.58 | 481.94 | 0.73 |
| ASV49 | 382.54 | 309.63 | 0.66 |
| ASV100 | 327.25 | 147.06 | 0.65 |
| ASV60 | 426.17 | 93.44 | 0.65 |
| ASV38 | 429.58 | 245.75 | 0.6 |
| ASV23 | 571.79 | 500.31 | 0.59 |
| ASV24 | 563.71 | 264.38 | 0.59 |
| ASV20 | 458.67 | 437.88 | 0.52 |
| ASV76 | 272.58 | 257.94 | 0.51 |
| ASV19 | 631.21 | 539 | 0.47 |
| ASV37 | 276.33 | 173.75 | 0.47 |
| ASV41 | 355.67 | 174.63 | 0.43 |
| ASV22 | 416.25 | 378.56 | 0.4 |
| ASV72 | 227.33 | 69.94 | 0.39 |
| ASV42 | 255.17 | 199 | 0.37 |
| ASV40 | 127.83 | 118 | 0.35 |
| ASV104 | 166.63 | 154.25 | 0.34 |
| ASV44 | 123.75 | 110.19 | 0.34 |
| ASV21 | 478.79 | 418.69 | 0.34 |
| ASV64 | 197.58 | 196 | 0.32 |
| ASV61 | 213.04 | 98.94 | 0.32 |
| ASV68 | 269.08 | 137.63 | 0.32 |
| ASV111 | 157.67 | 131.94 | 0.31 |
| ASV28 | 445.79 | 367.06 | 0.31 |
| ASV85 | 142.25 | 136.63 | 0.28 |
| ASV27 | 383.63 | 347.13 | 0.28 |
| ASV92 | 171.88 | 105.56 | 0.28 |
| ASV66 | 208.79 | 134.63 | 0.28 |
| ASV63 | 249.13 | 128.31 | 0.28 |
| ASV122 | 146.33 | 77.31 | 0.27 |
| ASV102 | 175.38 | 97.88 | 0.27 |
| ASV86 | 177.17 | 87.81 | 0.25 |
| ASV83 | 221.71 | 103.25 | 0.25 |
| ASV48 | 237.63 | 221.63 | 0.24 |
| ASV142 | 141.5 | 76.63 | 0.24 |
| ASV81 | 171.42 | 85.81 | 0.24 |
| ASV75 | 214.79 | 179.75 | 0.22 |
| ASV54 | 115.71 | 55.19 | 0.22 |
| ASV59 | 268.21 | 178.69 | 0.21 |

| B | Non-Severe cohort | Severe cohort |  |
| --- | --- | --- | --- |
| Species | Av.Abund | Av.Abund | Contrib% |
| ASV3 | 2075.25 | 3085.44 | 4.9 |
| ASV2 | 1741.88 | 2484.56 | 3.49 |
| ASV5 | 1549.75 | 2043.06 | 2.46 |
| ASV10 | 1008.21 | 1506.75 | 2.42 |
| ASV9 | 932.38 | 1757.06 | 2.39 |
| ASV7 | 968.25 | 1388.69 | 1.87 |
| ASV13 | 689.04 | 1246.5 | 1.79 |
| ASV16 | 748.5 | 922 | 1.24 |
| ASV18 | 503.21 | 598.31 | 1.11 |
| ASV30 | 425.79 | 605 | 1 |
| ASV32 | 378.46 | 565.94 | 0.91 |
| ASV15 | 513.13 | 660.75 | 0.91 |
| ASV34 | 311.63 | 622.81 | 0.85 |
| ASV29 | 299.58 | 525.69 | 0.7 |
| ASV46 | 328.83 | 384.19 | 0.65 |
| ASV36 | 346.33 | 352.44 | 0.58 |
| ASV35 | 340.17 | 351.94 | 0.56 |
| ASV33 | 224.5 | 296.56 | 0.54 |
| ASV62 | 246.04 | 275.06 | 0.53 |
| ASV47 | 113.67 | 352.5 | 0.5 |
| ASV52 | 264.25 | 305.19 | 0.48 |
| ASV45 | 275.04 | 312.94 | 0.48 |
| ASV17 | 189.13 | 248.19 | 0.47 |
| ASV55 | 186.13 | 341.13 | 0.46 |
| ASV57 | 157.21 | 277.81 | 0.44 |
| ASV90 | 126.58 | 243.75 | 0.41 |
| ASV67 | 195.08 | 203.56 | 0.37 |
| ASV56 | 25.63 | 215.63 | 0.36 |
| ASV53 | 81.96 | 186.94 | 0.36 |
| ASV78 | 164.63 | 209.94 | 0.34 |
| ASV96 | 63.67 | 205.63 | 0.32 |
| ASV212 | 54.92 | 150.06 | 0.29 |
| ASV43 | 75.08 | 171.19 | 0.28 |
| ASV87 | 96.38 | 165.75 | 0.28 |
| ASV101 | 110.13 | 133 | 0.28 |
| ASV94 | 112.54 | 184.75 | 0.27 |
| ASV58 | 188.17 | 252.69 | 0.27 |
| ASV25 | 416.25 | 433.13 | 0.27 |
| ASV124 | 63.08 | 155.69 | 0.26 |
| ASV117 | 76.25 | 135.13 | 0.26 |
| ASV105 | 114.79 | 134.81 | 0.23 |
| ASV39 | 71.17 | 90.75 | 0.23 |
| ASV84 | 40.71 | 150.19 | 0.22 |
| ASV126 | 71.88 | 141.19 | 0.22 |
| ASV50 | 60.83 | 91.88 | 0.22 |
| ASV160 | 24.5 | 114.44 | 0.21 |
| ASV99 | 90.63 | 156.69 | 0.21 |
| ASV134 | 47.42 | 102.69 | 0.21 |
| ASV106 | 88.38 | 128.19 | 0.2 |
